# Measurement of Oxidative Stress Index in Seminal Plasma Can Predict In Vivo Fertility of Liquid-Stored Porcine Artificial Insemination Semen Doses

**DOI:** 10.3390/antiox10081203

**Published:** 2021-07-27

**Authors:** Isabel Barranco, Camila P. Rubio, Asta Tvarijonaviciute, Heriberto Rodriguez-Martinez, Jordi Roca

**Affiliations:** 1 Department of Veterinary Medical Sciences, University of Bologna, 40064 Ozzano dell’Emilia, Bologna, Italy; isabel.barranco@unibo.it; 2 Department of Animal and Food Science, School of Veterinary Science, Universitat Autònoma de Barcelona, 08193 Cerdanyola del Vallès, Barcelona, Spain; camila.peres@uab.cat; 3 Interdisciplinary Laboratory of Clinical Analysis Interlab-UMU, Faculty of Veterinary Medicine, Regional Campus of International Excellence ‘Campus Mare Nostrum’, University of Murcia, 30100 Murcia, Spain; asta@um.es; 4 Department of Medicine and Animal Surgery, Faculty of Veterinary Medicine, University of Murcia, 30100 Murcia, Spain; 5 Department of Biomedical & Clinical Sciences (BKV), BKH/Obstetrics & Gynaecology, Faculty of Medicine and Health Sciences, Linköping University, 58185 Linköping, Sweden; heriberto.rodriguez-martinez@liu.se

**Keywords:** in vivo fertility, oxidative stress index, pig, seminal plasma, sperm quality

## Abstract

The study evaluated the relation between the oxidative stress index (OSI) in porcine seminal plasma (*n* = 76) with sperm resilience and in vivo fertility (farrowing rate and litter size of 3137 inseminated sows) of liquid-stored artificial insemination (AI) semen doses. The OSI was assessed as the ratio of advanced oxidation protein products to Trolox-equivalent antioxidant capacity, both measured using an automated analyzer. Sperm motility (computer-assisted sperm analyzer) and viability (flow cytometry) were evaluated in semen AI-doses at 0 and 72 h of storage at 17 °C. Sperm resilience was defined as the difference between storage intervals. Semen AI-doses were hierarchically clustered as having high, medium and low seminal OSI (*p* < 0.001) with those of low displaying higher resilience (*p* < 0.01). Boars were hierarchically clustered into two groups (*p* < 0.001) as having either positive or negative farrowing rate and litter size deviation; the negative one showing higher seminal OSI (*p* < 0.05). In sum, seminal OSI was negatively related to sperm motility and the in vivo fertility of liquid-stored boar semen AI-doses, with the receiver operating characteristic curve presenting seminal OSI as a good predictive biomarker of in vivo fertility of AI-boars (area under the curve: 0.815, *p* < 0.05).

## 1. Introduction

The aerobic metabolism of spermatozoa generates reactive oxygen species (ROS) which, in controlled amounts, are essential for sperm physiological processes, including capacitation, acrosome reaction, sperm–oocyte interaction, and further subsequent implantation and embryo development [1,2]. However, when ROS generation is excessive beyond the control of seminal plasma antioxidant mechanisms, spermatozoa experience oxidative stress (OS), which impairs their functionality and even leads to their death [2]. Specifically, this imbalance between ROS and antioxidants induces the peroxidation of lipids of the sperm plasma membrane (lipid peroxidation, LPO), resulting in a loss of sperm motility and membrane fluidity and integrity [3]. Moreover, the highly reactive molecules produced as result of LPO are also capable of causing sperm DNA damage, which have harmful effects on their fertilizing ability and subsequent embryo development [4].

Boar spermatozoa are especially vulnerable to OS due to their limited antioxidant defense mechanism in their cytoplasm [5] and the high concentration of polyunsaturated fatty acids in their plasma membrane [6], which, due to their double bonds, makes them particularly sensitive to ROS-induced damage mediated by LPO [2]. In this scenario, seminal plasma antioxidants play an essential role in scavenging excessive ROS and thus avoiding OS in spermatozoa [5,7]. Recent studies in this realm have reported that boar seminal plasma, a complex fluid mainly secreted by accessory sex glands, is endowed with a plethora of antioxidants (enzymatic and non-enzymatic), playing an essential role in sperm functional performance [8,9,10,11,12].

Artificial insemination (AI) is the most applied biotechnology for pig breeding in pork-producing countries, and is a key tool for genetic and production improvement [13,14]. Liquid storage at 17 °C is still the most widely used method for preserving AI-doses of pig semen, as it is easy to apply and preserves sperm functionality for 3–5 days, which is plenty of time for successful use in production farms [14]. Semen extenders play a key role in this success and their enrichment with additives having antioxidant capacity is encouraged with the ultimate purpose of minimizing potential OS and thus extending sperm functional life [13]. However, it should be noted that an excess of antioxidants may also lead to OS, induced by the “antioxidant paradox”, or reductive stress, decreasing the ROS levels required for sperm physiological function [15]. For this reason, the maintenance of an adequate balance between ROS and antioxidants levels is essential for optimal sperm function [2].

Measurement of the OS level in semen AI-doses has not yet been performed. This is even though it should be standard practice in AI-centers as it is in human andrology laboratories. This measurement would improve the current understanding of the causes of the low sperm performance of some semen AI-doses and would also be an objective criterion to determine the value of exogenous antioxidants in semen extenders. Oxidative stress in semen samples is usually assessed by the direct measurement of ROS (e.g., using the d-ROM test or by flow cytometry) or products resulting from ROS attack (e.g., LPO markers) [16]. These tests have some drawbacks, as they do not consider seminal plasma antioxidants that scavenge ROS, minimizing their possible damaging effects. Thus, laboratory tests able to measure the balance between oxidants and antioxidants in seminal plasma would be more objective to determine OS in semen samples. Among these tests would be the oxidative stress index (OSI); a quick, easy and inexpensive technique to accurately display oxidant/antioxidant ratio in biological samples [17]. In humans, seminal OSI values were related to male infertility issues [18,19,20,21]. However, as far as we know, no studies have been conducted in livestock assessing OSI in seminal plasma and evaluating its relationship with sperm performance and male fertility. In this context, this study aimed to test the usefulness of seminal OSI measurements in pig AI-centers. For this purpose, the relationship of seminal OSI with (1) the resistance of spermatozoa from semen AI-doses to withstand liquid storage at 17 °C for 72 h, and (2) the in vivo fertility outcomes of these liquid-stored semen AI-doses were evaluated. 

## 2. Materials and Methods

### 2.1. Reagents and Media

All reagents and media were purchased from Merck (KgaA, Darmstadt, Germany) and fluorochromes from Molecular Probes Europe BV (Leiden, The Netherlands), unless otherwise indicated. 

### 2.2. Boars, Ejaculates and Seminal Plasma Samples

Healthy, mature (1.5–3 years of age) and fertile boars of different breeds (Duroc, Landrace, Large White, and Pietrain) underwent regular semen collection to produce commercial semen AI-doses were used in the study. The boars belonged to the company AIM Ibérica (Topigs Norsvin Spain SLU), which fulfilled the Spanish (ES300130640127, August 2006) and European (ES13RS04P, July 2012) directions for animal health and welfare and ejaculate collection. All boars were housed in an AI-center (Calasparra, Murcia, Spain) in individual pens with controlled temperature (15–25 °C) and light (16 h/per day, artificial plus natural).

Entire ejaculates were collected from these AI-boars using a semi-automatic semen collection method (Collectis^®^, IMV Technologies, L’Aigle, France) at a frequency of two collections per week. Only ejaculates that satisfied the sperm quality requirements to produce commercial semen AI-doses (namely sperm concentration ˃200 × 10^6^ sperm/mL, sperm motility ˃70% and morphologically normal sperm ˃75%) were included in the study. 

For seminal plasma harvesting, entire ejaculates were twice centrifugated (1500× *g* for 10 min at room temperature (Rotofix 32A, Hettich Centrifuge UK, Newport Pagnell, Buckinghamshire, England, UK)), immediately after their collection. The resultant supernatants (seminal plasma samples) were analyzed in an Eclipse E400 microscope (Nikon, Tokyo, Japan) to assess it was sperm-free. Then, seminal plasma samples were sent in insulated containers (15–17 °C) to the Animal Andrology Laboratory of the Veterinary Teaching Hospital of the University of Murcia, where they arrived within three hours of ejaculate collection. Once in the laboratory, seminal plasma samples were immediately stored at −80 °C (Ultra Low Freezer; Haier Inc., Qingdao, China) until OSI measurements. 

### 2.3. Oxidative Stress Index Measurement in SP

Advanced oxidation protein products (AOPPs) and the Trolox equivalent antioxidant capacity (TEAC) of four seminal plasma samples were measured to calculate the OSI. Both assessments, AOPP and TEAC, were performed using an automated biochemistry analyzer (Olympus AU600 Automatic Chemistry Analyzer, Olympus Europe GmbH, Germany) and displayed an intra- and inter-assay coefficient variations ˂10%, showing linearity in serial dilutions. Each measurement was performed per duplicate in each seminal plasma sample.

The AOPPs, as markers of oxidant-mediated protein damage, were measured following the procedure described by Witko-Sarsat et al. [22], adapted to porcine seminal plasma. Briefly, 10 µL of the seminal plasma sample was mixed with 160 µL of 0.074 M potassium iodide and 25 µL of acetic acid (50%) and the change in absorbance was measured at 340 nm after incubation at 37 °C for 40 s. This assay was calibrated using chloramine-T (0–500 µM) and the results were expressed in μmol/L of chloramine-T equivalents. 

The TEAC, as a marker of total antioxidant capacity, was measured following the previously described method [23], and previously adapted to porcine seminal plasma samples. This assay was based on the ability of the non-enzymatic antioxidants to scavenge radical 2, 2′-Azino-bis(3-ethylbenzothiazoline-6-sulfonic acid) (ABTS) enzymatically pre-generated using an acid medium. Briefly, 12 µL of seminal plasma sample were mixed with 40 mmol/L acetate buffer (reagent 1) and with 2 mmol/L H_2_O_2_ and 10 mmol/L ABTS in a 30 mmol/L acetate buffer solution (reagent 2). The change in absorbance was measured at 600 nm. A concentration between 0.0 and 2.0 mmol/L of 6-hydroxy-2,5,7,8-tetramethylchromane-2-carboxylic acid (Trolox) was used to calibrate the assay and the results were expressed as mmol/L Trolox equivalent.

The OSI value was calculated using the formula described by Venturini et al. [24]: OSI (arbitrary unit) = AOPP (μmol/L) × 100 /TEAC (μmol/L). 

### 2.4. Assessment of Sperm Quality

Sperm quality parameters were assessed in extended semen samples (30 × 10^6^ sperm/mL), in terms of (1) sperm motility (total and progressive) and (2) sperm viability (integrity of plasma and acrosomal sperm membranes). Sperm motility was assessed using a computer-assisted sperm analyzer (ISASV1^®^, Proiser R+D S.L., Paterna, Spain). For that, semen samples (5 μL) were deposited in a pre-warmed (38 °C) Makler chamber (Sefi Medical Instruments, Haifa, Israel) and ten fields (˃600 spermatozoa/sample) were microscopically assessed. Spermatozoa with an average path velocity ˃20 μm/s were classified as motile and those with a straight-line velocity ˃40 μm/s as displaying progressive motility, and both were expressed as percentage. Sperm viability was assessed using a flow cytometry (BD FACS Canto II flow cytometer, Becton Dickinson & Company, Franklin Lakes, NJ, USA). For that, semen samples (100 μL) were incubated at 37 °C for 10 min (Sanyo MIR-153 incubator, Gemini BV, Apeldoorn, The Netherlands) with 3 μL of Hoechst 33342 (H-42; 0.05 mg/mL in phosphate buffered saline (PBS)), 2 μL of propidium iodide (PI, 0.5 mg/mL in PBS) and 2 μL fluorescein-conjugated peanut agglutinin (PNA-FITC, 100 μg/mL in PBS). Thereafter, samples were diluted in 400 μL of PBS for subsequent flow cytometric analysis. The sperm that exhibited intact plasma and acrosome membranes (H-42-positive/PI-negative/PNA-FITC-negative) were recorded as viable and results were expressed in percentage. A total of three technical replicates (with 10,000 H-42-positive events per each replicate) were performed.

### 2.5. Experimental Design

The experiments were authorized by the Bioethics Committee of the University of Murcia (research code: 639/2012).

#### 2.5.1. Experiment 1: Relationship between Seminal OSI and the Sperm Quality of Semen AI-Doses Stored at 17 °C for 72 h

Fifty-eight entire ejaculates (one ejaculate per boar) were collected and split into two aliquots: (1) the first aliquot was used to harvest seminal plasma (as detailed above) and stored at −80 °C until OSI measurement; (2) the second aliquot was extended in Beltsville Thaw Solution (BTS, Kubus, Las Rozas Madrid, Spain) alike an semen AI-dose (30 × 10^6^ sperm/mL), send to the Animal Andrology Laboratory of the Veterinary Teaching Hospital of the University of Murcia, where were stored at 17 °C during 72 h, assessing sperm quality (motility and viability) on arrival (0 h) and at 72 h of storage. Sperm resilience was defined as the difference between the percentages of sperm quality parameters measured at the 0 and 72 h storage time interval.

#### 2.5.2. Experiment 2: Relationship between Seminal OSI and In Vivo Fertility of Semen AI-Doses Stored at 17 °C

Eighteen Landrace and Large White boars included in a commercial AI-program provided ejaculates. For OSI measurements, seminal plasma samples were harvested from four ejaculates per boar collected over one year, at a rate of one ejaculate every four months. Ejaculates collected during the same year were extended in Biosem+ (Magapor, Ejea de los Caballeros, Zaragoza, Spain) at the rate of 30 × 10^6^ sperm/mL to produce AI-doses of 40 mL (1200 × 10^6^ total sperm) that were stored at 17 °C and used for intra-uterine AI (twice per estrus) of 3137 Landrace and Large White multiparous sows (˃100 sows inseminated per boar). These sows were housed in different Spanish farms subjected to same standard management, including two inseminations per estrus. The fertility of the inseminated sows was recorded in terms of: (1) farrowing rates (%): number of sows farrowed/number of sows inseminated × 100; (2) litter size: total number (alive plus dead) of piglets born per litter; and (3) a fertility index, defined as the total number of piglets born as a proportion of the number of sows inseminated. 

### 2.6. Statistical Analysis

Data were analyzed with the statistical software packages IBM SPSS Statistics 24.0 (IBM, Armonk, NY, USA) and GraphPad Prism 9.1.2 (GraphPad Software, Inc., La Jolla, CA, USA). Shapiro–Wilk test was conducted to assess the assumption of normality of the dataset. In the first experiment, Pearson correlation coefficients between SP-OSI and sperm parameters were calculated. Then, a hierarchical cluster analysis was carried out in order to classify ejaculates into three groups according to the seminal OSI (low, medium or high). A Kruskal–Wallis test, followed by Dunn’s post hoc test for multiple comparisons, was performed to evaluate putative differences on sperm quality parameters (sperm resiliency) among the three seminal OSI groups. In the second experiment, fertility records were subjected to a multivariate statistical model [25], in order to identify the direct boar effect for each fertility parameter (farrowing rate and litter size). Fertility data were expressed as deviation from the average fertility for the breed. Pearson correlation coefficients between seminal-OSI and fertility outcomes were calculated. Then, a hierarchical cluster analysis was carried out to classify the boars into two groups displaying negative or positive deviations in farrowing rate and litter size. A Mann–Whitney test was conducted to evaluate the putative differences on seminal OSI values between the two groups. A receiver operating characteristic (ROC) curve was performed to evaluate the predictive ability of the seminal OSI to discriminate between AI-boars with higher or lower fertility index (OSI threshold was 3). Areas under the ROC curve (AUC) and cut-off values were calculated by the program. Discrimination strength was measured by the AUC value following criteria: 1.00–0.90 (excellent), 0.90–0.80 (good), 0.80–0.70 (fair), 0.70–0.60 (poor), 0.60–0.50 (fail) and ˂0.50 (no discriminating). Significant statistical differences were defined from *p* < 0.05. 

## 3. Results

### 3.1. Experiment 1: Relationship between Seminal OSI and Sperm Quality of Semen AI-Doses Stored at 17 °C for 72 h

**Figure 1:**
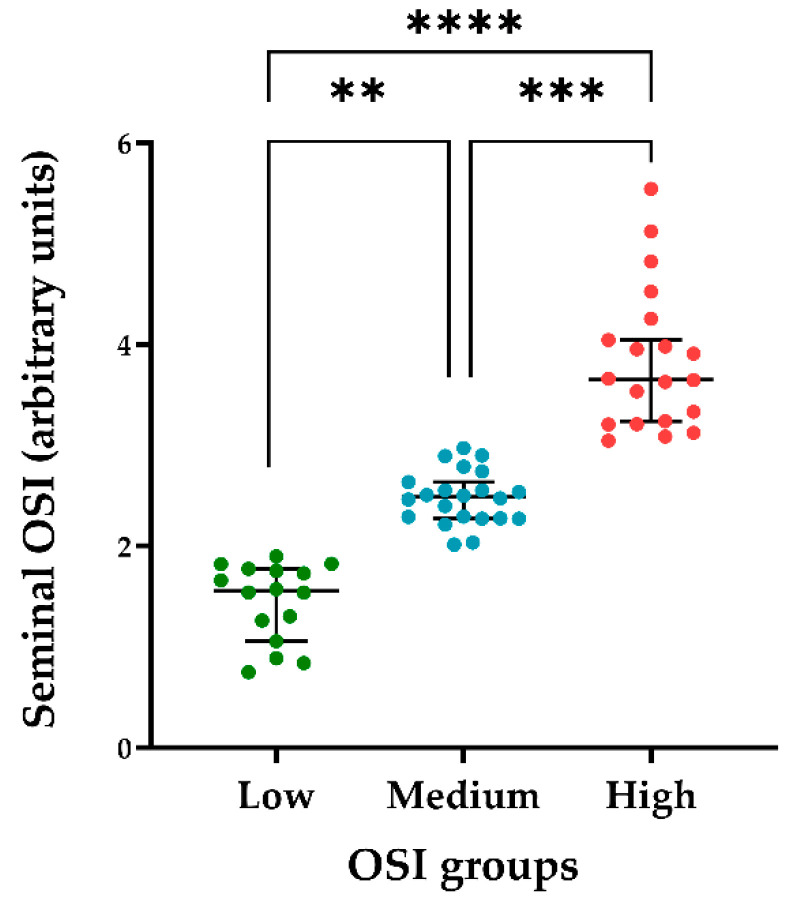
Values of oxidative stress index (OSI) in seminal plasma samples (*n* = 58) from porcine entire ejaculates (*n* = 58; one per boar). The ejaculates were clustered according to their seminal OSI values (hierarchical clustering, *p* < 0.01) as having low (between 0.75 and 1.89; *n* = 16), medium (between 2.01 and 2.91; *n* = 22) or high (between 3.04 and 5.54; *n* = 20) OSI values. The line indicates the median, the whiskers extend to the 5th and 95th percentiles, and dots represent the seminal OSI values. **** *p* < 0.00001; *** *p* < 0.0001; ** *p* < 0.001.

The OSI in the 58 seminal plasma samples ranged from 0.75 to 5.54. The Pearson correlation coefficients showed that seminal OSI was not related to the sperm quality parameters of semen AI-doses assessed at either 0 or 72 h of storage at 17 °C (Supplementary Figure S1). Therefore, the ejaculates were grouped according to their seminal OSI by hierarchical clustering, and three groups were generated (*p* < 0.01) as having either low (between 0.75 and 1.89; *n* = 16), medium (between 2.01 and 2.91; *n* = 22) or high (between 3.04 and 5.54; *n* = 20) seminal OSI (Figure 1). 

**Figure 2:**
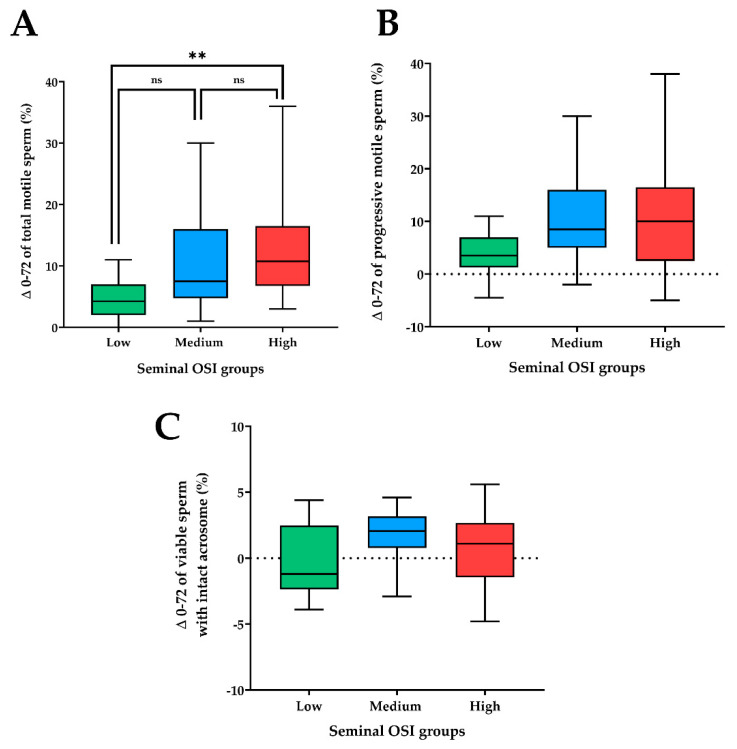
Box-whisker plot showing the differences (sperm resilience) in the percentages of (**A**) total motile sperm; (**B**) progressive motile sperm; and (**C**) viable sperm with intact acrosome between both evaluation time-points (0 and 72 h of storage at 17 °C) of artificial insemination semen doses (*n* = 58) of boar ejaculates clustered into three groups (hierarchical clustering, *p* < 0.01) according to the oxidative stress index (OSI) of seminal plasma. Low seminal OSI ranged from 0.75 to 1.89 (16 semen doses), medium from 2.01 to 2.91; (22 semen doses) and high from 3.04 to 5.54 (20 semen doses). Boxes enclose the 25th and 75th percentiles, whiskers extend to the 5th and 95th percentiles and the line indicates the median. ** *p* < 0.001; ns: not significant.

Sperm motility resilience differed among seminal OSI groups (*p* < 0.01). Semen AI-doses from ejaculates with low seminal OSI showed less differences in total motility between 0 and 72 h of storage at 17 °C than those with high seminal OSI (Figure 2A). The differences ranged from 0 to 11% in the semen AI-doses from ejaculates classified with low seminal OSI values (only 2 of the 16 semen AI-doses had a difference ≥10%), and from 3 to 36% in those classified with high seminal OSI values (13 of the 20 semen AI-doses had a difference ≥10%). The differences in the percentages of progressive motile and viable spermatozoa with intact acrosome were similar among semen AI-doses of the three seminal OSI groups (Figure 2B,C).

### 3.2. Experiment 2: Relationship between Seminal OSI and In Vivo Fertility of Semen AI-Doses Stored at 17 °C 

**Figure 3:**
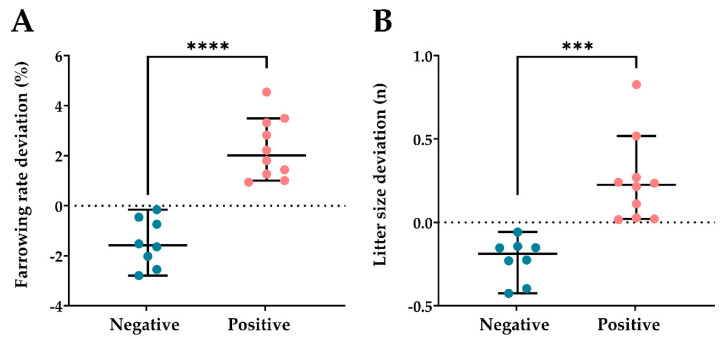
Distribution by hierarchical cluster analysis (*p* < 0.001) of the 18 boars used in artificial insemination programs into two groups for having (**A**) negative (between −2.79 and −0.16; *n* = 8) or positive (between +0.94 and +4.54; *n* = 10) farrowing rate deviations; and (**B**) negative (between −0.43 and −0.05; *n* = 8) or positive (between +0.01 and +0.83; *n* = 10) litter size deviations. The line indicates the median, the whiskers extend to the 5th and 95th percentiles, and the dots represent the boars. **** *p* < 0.0001; *** *p* < 0.001.

**Figure 4:**
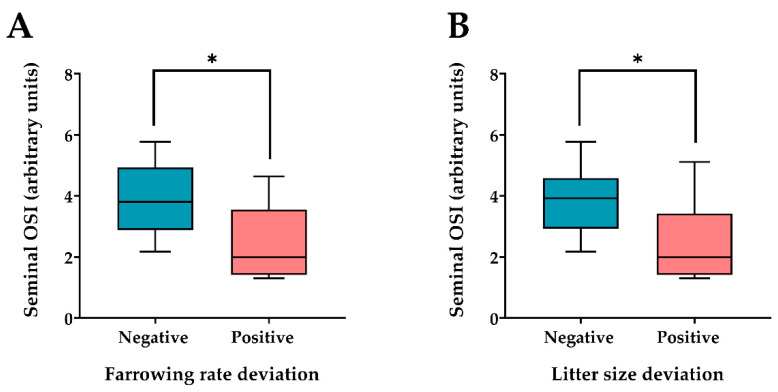
Box-whisker plot showing oxidative stress index (OSI) values assessed in the seminal plasma of ejaculates collected from artificial insemination boars hierarchically (*p* < 0.001) grouped as showing (**A**) negative (ranging from −2.79 to −0.16; *n* = 8) or positive (ranging from +0.94 to +4.54; *n* = 10) farrowing rate deviations and (**B**) negative (ranging from −0.43 to −0.05; *n* = 8) or positive (ranging from +0.01 to +0.83; *n* = 10) litter size deviations. Boxes enclose the 25th and 75th percentiles, the line indicates the median and the whiskers extend to the 5th and 95th percentiles. * Indicates significant differences (*p* < 0.05) in seminal OSI values between groups.

Fertility data were statistically corrected to determine the direct effect of the boar and the results for each boar were recorded as the deviation from the mean of boar population of the same genetic line. The deviation ranged between +4.54 and −2.79 for the farrowing rate and between +0.83 and −0.43 for the number of piglets born per farrowing. The OSI values of the 72 seminal plasma samples (four per boar) ranged from 0.81 to 7.42. The correlation coefficient of Pearson evidenced that seminal OSI was weakly related with farrowing rate deviation (*p* < 0.05; R = −0.54) and was not related with litter size deviation (*p* > 0.05; R = −0.34). Then, the 18 AI-boars included in this study were hierarchically clustered (*p* < 0.001) into two groups as having either a negative (ranging from −2.79 to −0.16; *n* = 8) or positive (ranging from +0.94 to +4.54; *n* = 10) farrowing rate deviation (Figure 3A). Similarly, the boars were also clustered into two groups as having either negative (ranging from −0.43 to −0.05; *n* = 8) or positive (ranging from +0.01 to +0.83; *n* = 10) litter size deviation (Figure 3B). The seminal OSI values were different (*p* < 0.05) between the two groups of boars for both the farrowing rate and litter size. Boars with negative deviations showed higher (*p* < 0.05) seminal OSI than those with positive deviations (Figure 4A,B).

**Figure 5:**
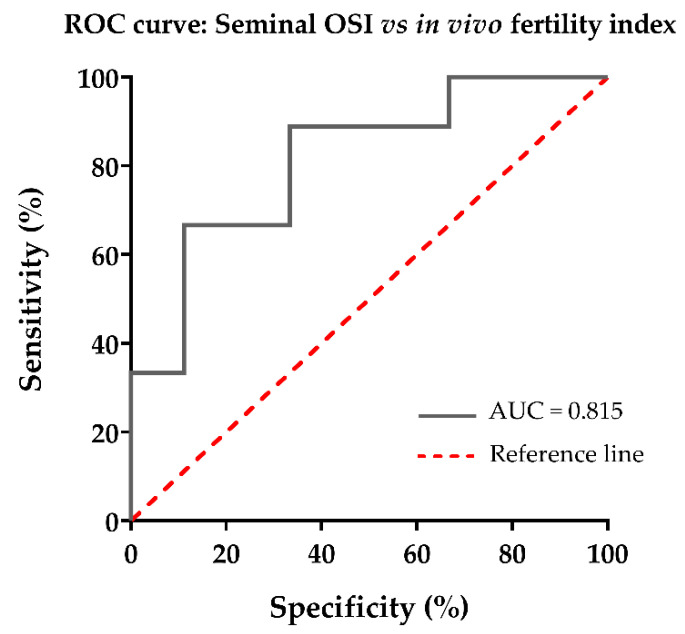
Nonparametric receivers operating characteristic (ROC) curve showing the ability of oxidative stress index (OSI) assessed in seminal plasma to predict in vivo fertility index of boars whose semen, as liquid-stored semen doses (17 °C), was used for artificial insemination. AUC: area under the ROC curve.

Like the first experiment, the boars were grouped according to their seminal OSI (mean of four seminal plasma samples from four different ejaculates collected over a year) by hierarchical clustering, and two groups were generated (*p* < 0.01) with either a low (*n* = 9) or high (*n* = 9) seminal OSI (OSI threshold was 3). The ROC-curve showed that the seminal OSI had good discriminating ability to predict the in vivo fertility of AI-boars, measured in terms of fertility index (AUC = 0.815; *p* < 0.05) (Figure 5).

## 4. Discussion

As far as we are aware, this is the first report conducted in a livestock species measuring OSI in seminal plasma and assessing its putative influence on sperm quality and the fertility of semen AI-doses. The results showed that seminal OSI values were negatively related with the sperm motility of semen AI-doses storage at 17 °C for 72 h, as well as in vivo fertility, in terms of farrowing rates and litter size. 

OSI is not currently included in the battery of tests used for ejaculate selection in AI centers for livestock species, despite the fact it is well known that fresh semen samples generate ROS that impair sperm functionality [26]. In pigs, seminal ROS levels increase during semen liquid storage, compromising the functional lifespan of stored spermatozoa, including their fertilizing ability [13,26,27,28]. In addition, the extension of semen prior to liquid storage lowers the concentration of seminal plasma antioxidants. The increase in ROS together with the low antioxidant concentration in semen AI-doses results in both an unbalanced ROS: antioxidants ratio and negative influence on the sperm performance. Supplementing semen extender with antioxidants is one of the most accepted options to avoid this undesirable scenario [7,13]. However, this supplementation often has a detrimental effect on sperm functionality, especially when unsuitable high concentrations are used [27,29,30,31,32], as they lead to a complete inhibition of ROS, which negatively impairs key sperm functions such as capacitation [33,34]. Taken together, these studies support the argument that measuring seminal OSI could be a good option to improve the selection of ejaculates used for AI. Different assays have been proposed in the literature to evaluate the ROS/antioxidant ratio in human semen (reviewed by Agarwal et al., [35]). The oxidation–reduction potential, based on the measurement of the potential of electrons to change from one chemical species to another, and OSI or ROS-TAC score, a measure of oxidation/antioxidation ratio, will be among the best-known direct assays [35]. To calculate OSI, both the oxidant and antioxidant capacities can be measured using different procedures. For instance, ferric reducing antioxidant potential, total antioxidant activity or TEAC for measuring antioxidant capacity; and total oxidative capacity, total oxidative status or AOPP for measuring oxidative capacity [18,19,20,21]. In porcine, Wang et al. [27] measured OSI in the supernatant of extended liquid-stored semen samples using the ratio of the total oxidative status to TEAC. Although these assays would be suitable to calculate OSI, Venturini et al. [24] suggested that the AOPP/TEAC ratio could be a better indicator for OS. Accordingly, we opted for this test because it was simple, quick to perform and rather inexpensive, the latter reason being an important requirement for porcine insemination centers due to their narrow profit margins. 

The seminal OSI values varied widely between ejaculates and between boars. This variation was expected, since both ROS levels and antioxidant concentrations in seminal plasma also vary widely between ejaculates and boars [8,9,10,11,12]. Unfortunately, we cannot discuss whether the seminal OSI values are high or low since there are no previous studies that have evaluated this seminal plasma index in semen from porcine or other livestock species. The seminal OSI values were not related with the loss of sperm viability, measured in terms of plasma and acrosomal membrane integrity during liquid storage. In agreement with this finding, previous studies reported that this sperm parameter is little affected by the 72 h liquid storage of porcine semen [11,28,36,37]. However, seminal OSI values were related to the loss of total sperm motility during semen storage time. It has been extensively reported that an excess of ROS has detrimental effects on sperm motility [38,39]. The ROS:antioxidants unbalance would cause mitochondrial dysfunction, leading to adenosine triphosphate depletion, decreasing energy availability and thereby impairing sperm motility [40]. An excess of ROS also negatively influences the activity of some enzymes essential for sperm motility, such as glucose-6-phosphate dehydrogenase [41]. Studies conducted in pig semen have linked the loss of sperm motility in liquid-stored semen samples to an increase in malondialdehyde, one of the end-products of the ROS-induced peroxidation of sperm membrane lipids [26,42]. This, and other end-products of LPO of sperm membranes such as 4-hydroxynonenol, can also cause a decrease in sperm motility because they modify the permeability of the sperm plasma membrane and thus their ability to regulate intracellular ions [43]. 

The seminal OSI differed between boars with high and low fertility outcomes, for both farrowing rates and litter sizes. There are no previous studies evaluating the putative association between seminal OSI and in vivo fertility in livestock species. Human studies have reported the usefulness of seminal OSI in predicting male fertility, showing greater predictive ability than the measurements of ROS or total antioxidant capacity alone in discriminating between fertile and infertile men [18,19,20,21,44]. The negative relationship between seminal OSI and in vivo fertility would be related to the negative effect of seminal OSI on total sperm motility, since it is well-known that the percentage of total motile spermatozoa is positively related to in vivo fertility of liquid-stored pig semen doses [45]. In addition, the aforementioned peroxidation of sperm membrane lipids, associated with excessive ROS due to an unbalance between ROS and antioxidants, leads to a decrease in membrane fluidity, which is necessary for sperm–oocyte fusion and fertilization [46]. Excessive ROS levels could also adversely affect fertility by inducing sperm nuclear DNA fragmentation [39]. A high DNA fragmentation may reduce the fertilization rates, implantation, embryo development, increasing pregnancy loss [35]. In this regard, Boe–Hansen et al. [47] reported an increase in sperm nuclear DNA fragmentation in boar semen samples after 72 h of liquid storage. These authors also observed that the percentage of affected sperm varied significantly between ejaculates, which could be related to the differences in seminal OSI between ejaculates. 

Pig AI-centers are calling for the development of predictive tests to identify subfertile boars prior to their inclusion in AI-programs, as it is estimated that 5–7% of boars included in insemination programs are subfertile even though their ejaculates show good sperm quality [48]. Under field conditions, the two measures that define in vivo fertility in swine, namely farrowing rate and litter size, cannot be isolated from each other. Therefore, the fertility index, combining both measures, would be a more accurate measurement of boar in vivo fertility. The ROC-curve showed that seminal OSI had a good predicting ability. Accordingly, seminal OSI could be a good candidate as an in vivo fertility biomarker for boars included in artificial insemination programs.

This study has some limitations that should be considered in future studies. The first would be related to the sperm assessments. In this study, with the purpose of approaching the reality of the laboratories of swine AI-centers, we only evaluated those sperm parameters that are usually evaluated in AI-centers to select valid ejaculates for use in AI-programs. Including other sperm functional tests such as LPO and nuclear DNA fragmentation would help better understand the relationship between seminal OSI and sperm function. The second limitation would be related to the AI trial, which was performed under field conditions, also with the purpose of assessing the usefulness of measuring seminal OSI in the operating work context of AI-centers. Thus, the semen doses used in the AI-trial were those produced and marketed by the AI-center, using a semen extender of unknown public composition (Biosem+). The use of semen extenders of known elemental composition would exclude external factors that may influence fertility associated with semen extender components.

## 5. Conclusions

In conclusion, the seminal OSI was positively related with sperm motility loss in liquid-stored extended boar semen. In addition, seminal OSI was negatively related with the in vivo fertility of liquid-stored semen doses, measured in terms of farrowing rate and the litter size of sows receiving intra-uterine inseminations. The ROC-curve showed seminal OSI, an easy, quick, and inexpensive method, which could be a good predictive biomarker for in vivo fertility. Therefore, it could be economically profitable to include it in the battery of tests routinely used in pig AI-centers. The measurement of seminal OSI would help identify subfertile boars prior to their inclusion in AI-programs. It would also be useful in identifying ejaculates that would require antioxidant supplementation to retain functional sperm life during preservation.

## Data Availability

Data is contained within the article and Supplementary Materials.

## Supplementary Materials

The following are available online at www.mdpi.com/article/10.3390/antiox10081203/s1, Figure S1: Heat map showing Pearson’s correlation coefficients between seminal plasma oxidative stress index (OSI) and sperm quality parameters of porcine artificial insemination semen doses (semen AI-doses, *n* = 58). The semen quality parameters were assessed at 0 and 72 h of storage at 17 °C and the correlation coefficients between seminal OSI and the sperm resilience (difference in each sperm quality parameter between evaluation time-points: 0 and 72 h) was also assessed.

## References

[1] de Lamirande E., Jiang H., Zini A., Kodama H., Gagnon C. (1997). Reactive oxygen species and sperm physiology. Rev. Reprod..

[2] Bansal A.K., Bilaspuri G.S. (2010). Impacts of oxidative stress and antioxidants on semen functions. Vet. Med. Int..

[3] Aitken R.J., Gibb Z., Baker M.A., Drevet J., Gharagozloo P. (2016). Causes and consequences of oxidative stress in spermatozoa. Reprod. Fertil. Dev..

[4] Ribas-Maynou J., Yeste M. (2020). Oxidative stress in male infertility: Causes, effects in assisted reproductive techniques, and protective support of antioxidants. Biology.

[5] Strzezek J., Lapkiewicz S., Lecewicz M. (1999). A note on antioxidant capacity of boar seminal plasma. Anim. Sci. Pap. Rep..

[6] Cerolini S., Maldjian A., Surai P., Noble R. (2000). Viability, susceptibility to peroxidation and fatty acid composition of boar semen during liquid storage. Anim. Reprod. Sci..

[7] Bathgate R. (2011). Antioxidant mechanisms and their benefit on post-thaw boar sperm quality. Reprod. Domest. Anim..

[8] Barranco I., Tvarijonaviciute A., Perez-Patiño C., Parrilla I., Ceron J.J., Martinez E.A., Rodriguez-Martinez H., Roca J. (2015). High total antioxidant capacity of the porcine seminal plasma (SP-TAC) relates to sperm survival and fertility. Sci. Rep..

[9] Barranco I., Tvarijonaviciute A., Perez-Patino C., Alkmin D.V., Ceron J.J., Martinez E.A., Rodriguez-Martinez H., Roca J. (2015). The activity of paraoxonase type 1 (PON-1) in boar seminal plasma and its relationship with sperm quality, functionality, and in vivo fertility. Andrology.

[10] Barranco I., Tvarijonaviciute A., Perez-Patino C., Vicente-Carrillo A., Parrilla I., Ceron J.J., Martinez E.A., Rodriguez-Martinez H., Roca J. (2016). Glutathione Peroxidase 5 is expressed by the entire pig male genital tract and once in the seminal plasma contributes to sperm survival and in vivo fertility. PLoS ONE.

[11] Barranco I., Padilla L., Tvarijonaviciute A., Parrilla I., Martínez E.A., Rodriguez-Martinez H., Yeste M., Roca J. (2019). Levels of activity of superoxide dismutase in seminal plasma do not predict fertility of pig AI-semen doses. Theriogenology.

[12] Li J., Barranco I., Tvarijonaviciute A., Molina M.F., Martinez E.A., Rodriguez-Martinez H., Parrilla I., Roca J. (2018). Seminal plasma antioxidants are directly involved in boar sperm cryotolerance. Theriogenology.

[13] Yeste M. (2017). State-of-the-art of boar sperm preservation in liquid and frozen state. Anim. Reprod..

[14] Waberski D., Riesenbeck A., Schulze M., Weitze K.F., Johnson L. (2019). Application of preserved boar semen for artificial insemination: Past, present and future challenges. Theriogenology.

[15] Henkel R., Sandhu I.S., Agarwal A. (2019). The excessive use of antioxidant therapy: A possible cause of male infertility?. Andrologia.

[16] Robert K.A., Sharma R., Henkel R., Agarwal A. (2021). An update on the techniques used to measure oxidative stress in seminal plasma. Andrologia.

[17] Abuelo A., Hernández J., Benedito J.L., Castillo C. (2013). Oxidative stress index (OSi) as a new tool to assess redox status in dairy cattle during the transition period. Animal.

[18] Kolettis P.N., Sharma R.K., Pasqualotto F.F., Nelson D., Thomas A.J.J., Agarwal A. (1999). Effect of seminal oxidative stress on fertility after vasectomy reversal. Fertil. Steril..

[19] Sharma R.K., Pasqualotto F.F., Nelson D.R., Thomas A.J.J., Agarwal A. (1999). The reactive oxygen species-total antioxidant capacity score is a new measure of oxidative stress to predict male infertility. Hum. Reprod..

[20] Pasqualotto F.F., Sundaram A., Sharma R.K., Borges E.J., Pasqualotto E.B., Agarwal A. (2008). Semen quality and oxidative stress scores in fertile and infertile patients with varicocele. Fertil. Steril..

[21] Takeshima T., Usui K., Mori K., Asai T., Yasuda K., Kuroda S., Yumura Y. (2021). Oxidative stress and male infertility. Reprod. Med. Biol..

[22] Witko-Sarsat V., Friedlander M., Capeillère-Blandin C., Nguyen-Khoa T., Nguyen A.T., Zingraff J., Jungers P., Descamps-Latscha B. (1996). Advanced oxidation protein products as a novel marker of oxidative stress in uremia. Kidney Int..

[23] Rubio C.P., Tvarijonaviciute A., Caldin M., Hernández-Ruiz J., Cerón J.J., Martínez-Subiela S., Tecles F. (2018). Stability of biomarkers of oxidative stress in canine serum. Res. Vet. Sci..

[24] Venturini D., Simão A.N.C., Urbano M.R., Dichi I. (2015). Effects of extra virgin olive oil and fish oil on lipid profile and oxidative stress in patients with metabolic syndrome. Nutrition.

[25] Broekhuijse M.L.W.J., Sostaric E., Feitsma H., Gadella B.M. (2012). The value of microscopic semen motility assessment at collection for a commercial artificial insemination center, a retrospective study on factors explaining variation in pig fertility. Theriogenology.

[26] Kumaresan A., Kadirvel G., Bujarbaruah K.M., Bardoloi R.K., Das A., Kumar S., Naskar S. (2009). Preservation of boar semen at 18 degrees C induces lipid peroxidation and apoptosis like changes in spermatozoa. Anim. Reprod. Sci..

[27] Wang S., Sun M., Wang N., Yang K., Guo H., Wang J., Zhang Y., Yue S., Zhou J. (2018). Effects of L-glutamine on boar sperm quality during liquid storage at 17°C. Anim. Reprod. Sci..

[28] Barranco I., Padilla L., Perez-Patino C., Vazquez J.M., Martinez E.A., Rodriguez-Martinez H., Roca J., Parrilla I. (2019). Seminal plasma cytokines are predictive of the outcome of boar sperm preservation. Front. Vet. Sci..

[29] Zhang X.G., Liu Q., Wang L.Q., Yang G.S., Hu J.H. (2016). Effects of glutathione on sperm quality during liquid storage in boars. Anim. Sci. J..

[30] Zhang X.G., Li H., Wang L., Hao Y.Y., Liang G.D., Ma Y.H., Yang G.S., Hu J.H. (2017). The effects of different levels of superoxide dismutase in Modena on boar semen quality during liquid preservation at 17 °C. Anim. Sci. J..

[31] Li H., Zhang X.G., Fang Q., Liu Q., Du R.R., Yang G.S., Wang L.Q., Hu J.H. (2017). Supplemental effect of different levels of taurine in Modena on boar semen quality during liquid preservation at 17 °C. Anim. Sci. J..

[32] Yang K., Wang N., Guo H.T., Wang J.R., Sun H.H., Sun L.Z., Yue S.L., Zhou J.B. (2020). Effect of L-carnitine on sperm quality during liquid storage of boar semen. Asian-Australas. J. Anim. Sci..

[33] Aitken R.J. (2017). Reactive oxygen species as mediators of sperm capacitation and pathological damage. Mol. Reprod. Dev..

[34] O’Flaherty C. (2015). Redox regulation of mammalian sperm capacitation. Asian J. Androl..

[35] Agarwal A., Roychoudhury S., Bjugstad K.B., Cho C.-L. (2016). Oxidation-reduction potential of semen: What is its role in the treatment of male infertility?. Ther. Adv. Urol..

[36] Barranco I., Casao A., Perez-Patiño C., Parrilla I., Muiño-Blanco T., Martinez E.A., Cebrian-Perez J.A., Roca J. (2017). Profile and reproductive roles of seminal plasma melatonin of boar ejaculates used in artificial insemination programs. J. Anim. Sci..

[37] Mateo-Otero Y., Fernández-López P., Ribas-Maynou J., Roca J., Miró J., Yeste M., Barranco I. (2021). Metabolite profiling of pig seminal plasma identifies potential biomarkers for sperm resilience to liquid preservation. Front. Cell Dev. Biol..

[38] Agarwal A., Ikemoto I., Loughlin K.R. (1994). Relationship of sperm parameters with levels of reactive oxygen species in semen specimens. J. Urol..

[39] Agarwal A., Saleh R.A., Bedaiwy M.A. (2003). Role of reactive oxygen species in the pathophysiology of human reproduction. Fertil. Steril..

[40] de Lamirande E., Gagnon C. (1992). Reactive oxygen species and human spermatozoa. II. Depletion of adenosine triphosphate plays an important role in the inhibition of sperm motility. J. Androl..

[41] Aitken R.J., Fisher H.M., Fulton N., Gomez E., Knox W., Lewis B., Irvine S. (1997). Reactive oxygen species generation by human spermatozoa is induced by exogenous NADPH and inhibited by the flavoprotein inhibitors diphenylene iodonium and quinacrine. Mol. Reprod. Dev..

[42] Karunakaran M., Chakurkar E.B., Ratnakaran U., Naik P.K., Mondal M., Mondal A., Singh N.P. (2017). Characteristics of boar semen preserved at liquid state. J. Appl. Anim. Res..

[43] Baumber J., Ball B.A., Gravance C.G., Medina V., Davies-Morel M.C. (2000). The effect of reactive oxygen species on equine sperm motility, viability, acrosomal integrity, mitochondrial membrane potential, and membrane lipid peroxidation. J. Androl..

[44] Agarwal A., Makker K., Sharma R. (2008). Clinical relevance of oxidative stress in male factor infertility: An update. Am. J. Reprod. Immunol..

[45] Popwell J.M., Flowers W.L. (2004). Variability in relationships between semen quality and estimates of in vivo and in vitro fertility in boars. Anim. Reprod. Sci..

[46] Aitken R.J., Harkiss D., Buckingham D. (1993). Relationship between iron-catalysed lipid peroxidation potential and human sperm function. J. Reprod. Fertil..

[47] Boe-Hansen G.B., Ersbøll A.K., Greve T., Christensen P. (2005). Increasing storage time of extended boar semen reduces sperm DNA integrity. Theriogenology.

[48] Roca J., Broekhuijse M.L.W.J., Parrilla I., Rodriguez-Martinez H., Martinez E.A., Bolarin A. (2015). Boar differences in artificial insemination outcomes: Can they be minimized?. Reprod. Domest. Anim..

