# Supplementary material for: Measurement of Oxidative Stress Index in Seminal Plasma Can Predict In Vivo Fertility of Liquid-Stored Porcine Artificial Insemination Semen Doses
Source: Antioxidants (Basel). 2021 Jul 27;10(8):1203. doi: 10.3390/antiox10081203 (PMC8388916; doi:10.3390/antiox10081203)
Supplement: Supplementary file 1 [file antioxidants-10-01203-s001.zip › antioxidants-1304967-supplementary.pdf]

antioxidants

MDPI

Article

Measurement of Oxidative Stress Index in Seminal Plasma Can Predict In Vivo Fertility of Liquid-Stored Porcine Artificial Insemination Semen Doses

Isabel Barranco [1], Camila P Rubio [2,3], Asta Tvarijonaviciute [3,4], Heriberto Rodriguez-Martinez [5] and Jordi Roca [4,*]

1 Department of Veterinary Medical Sciences, University of Bologna, 40064 Ozzano dell'Emilia, Bologna, Italy; isabel.barranco@unibo.it
2 Department of Animal and Food Science, School of Veterinary Science, Universitat Autònoma de Barcelona, 08193 Cerdanyola del Vallès, Barcelona, Spain; camila.peres@uab.cat
3 Interdisciplinary Laboratory of Clinical Analysis Interlab-UMU, Faculty of Veterinary Medicine, Regional Campus of International Excellence 'Campus Mare Nostrum', University of Murcia, 30100 Murcia, Spain; asta@um.es
4 Department of Medicine and Animal Surgery, Faculty of Veterinary Medicine, University of Murcia, 30100 Murcia, Spain
5 Department of Biomedical & Clinical Sciences (BKV), BKH/Obstetrics & Gynaecology, Faculty of Medicine and Health Sciences, Linköping University, 58185 Linköping, Sweden; heriberto.rodriguez-martinez@liu.se
* Correspondence: roca@um.es

Supplementary Materials:

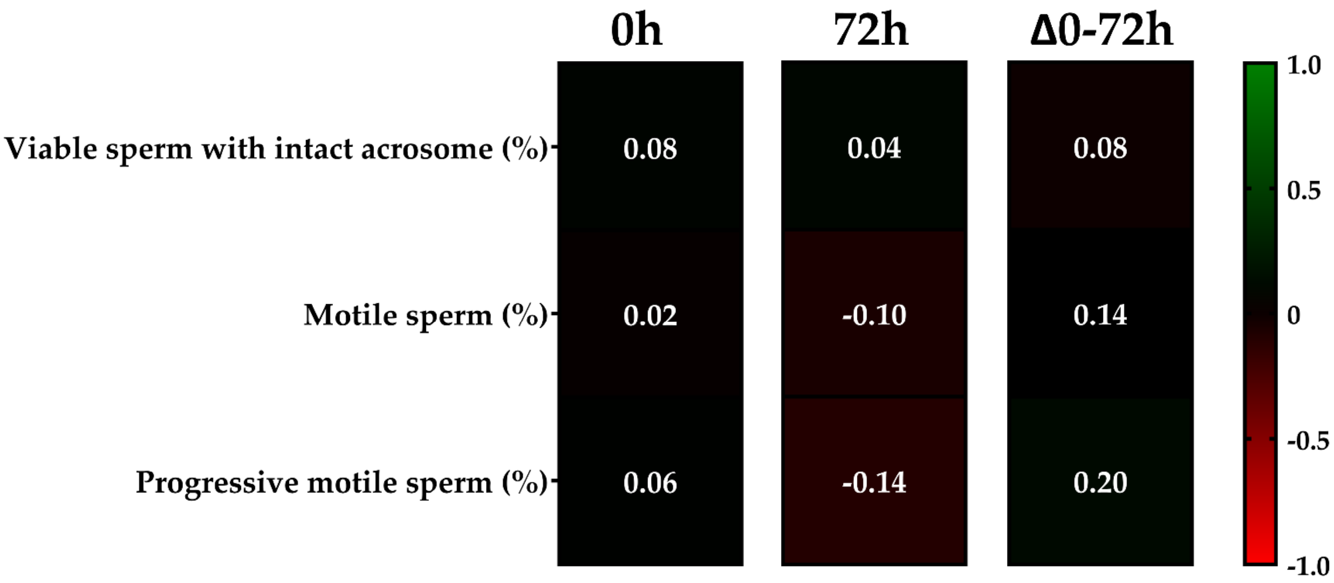

Figure S1. Heat map showing Pearson's correlation coefficients between seminal plasma oxidative stress index (OSI) and sperm quality parameters of porcine artificial insemination semen doses (semen AI-doses, $n = 58$). The semen quality parameters were assessed at 0 and 72 h of storage at 17 °C and the correlation coefficients between seminal OSI and the sperm resilience (difference in each sperm quality parameter between evaluation time-points: 0 and 72 h) was also assessed.
